# Vulnerability of vascular endothelium in lipopolysaccharide toxicity: effect of (acyl) carnitine on endothelial stability

**DOI:** 10.1155/S0962935193000705

**Published:** 1993

**Authors:** W. C. Hülsmann

**Affiliations:** Erasmus University Thorax Centre P.O. Box 1738 Rotterdam 3000 DR The Netherlands

## Abstract

The literature presented illustrates that lipopolysaccharide (LPS), from bacterial cell walls, induces tumour necrosis factor (TNF) synthesis in macrophages. TNF affects a number of cell types, amongst which are endothelial cells, within a few hours. Its injection has been shown to produce all symptoms of the toxic syndrome. In the present communication the vulnerability of endothelial cells will be stressed. These cells require carnitine not only for fatty acid oxidation but also for membrane protection and repair. As endothelial cells lose carnitine during hypoperfusion, it is speculated that the supply of carnitine during the early phase of LPS toxicity in rats might delay or avoid loss of endothelial functions. Earlier it was observed that hearts from rats, injected 3 h previously with LPS, showed strongly increased interstitial fluid production compared to hearts from control rats, even when TNF was present during a 3 h *in vitro* perfusion. It showed that LPS *in vivo* generates factors other than TNF, such as platelet activating factor (PAF), that are responsible for the increased capillary permeability.


Mediators of Inflammation 2, S21–S23 (1993)

### Vulnerability of vascular endothelium in lipopolysaccharide toxicity: effect of (acyl) carnitine on endothelial stability

W.C. Hülsmann

Erasmus University, Thorax Centre, P.O. Box 1738, 3000 DR Rotterdam, The Netherlands

CA Corresponding Author

**The literature presented illustrates that lipopolysaccharide (LPS), from bacterial cell walls, induces tumour necrosis factor (TNF) synthesis in macrophages. TNF affects a number of cell types, amongst which are endothelial cells, within a few hours. Its injection has been shown to produce all symptoms of the toxic syndrome. In the present communication the vulnerability of endothelial cells will be stressed. These cells require carnitine not only for fatty acid oxidation but also for membrane protection and repair. As endothelial cells lose carnitine during hypoperfusion, it is speculated that the supply of carnitine during the early phase of LPS toxicity in rats might delay or avoid loss of endothelial functions. Earlier it was observed that hearts from rats, injected 3 h previously with LPS, showed strongly increased interstitial fluid production compared to hearts from control rats, even when TNF was present during a 3 h *in vitro* perfusion. It showed that LPS *in vivo* generates factors other than TNF, such as platelet activating factor (PAF), that are responsible for the increased capillary permeability.**

**Key words:** Carnitine, Endothelium, LPS, PAF, TNF

### Introduction

The study of the pathophysiology of septic shock has gained momentum since the discovery that lipopolysaccharide (LPS) from the cell walls of Gram-negative bacteria, is mainly responsible for the toxic syndrome. LPS induces the synthesis of tumour necrosis factor (TNF) in macrophages, which has been isolated from these cells.[1] The structure of TNFα has been determined by cDNA cloning[2] and appeared identical to that of cachectin.[1] TNF causes cachexia and a state of shock.[3] *In vitro*, it is cytostatic or cytolytic for various tumour cell lines. It is not cytotoxic to normal cells.[3] TNF suppresses lipoprotein lipase,[4] an enzyme on macrophages and vascular endothelium of red muscle cells and fat cells for the hydrolysis of glycerides of plasma lipoproteins. TNF also affects other activities of endothelial cells, such as stimulation of procoagulant activity,[5] induction of phospholipase $A_2$[6] and inhibition of proteoglycan synthesis.[7] It enhances adhesion with neutrophils[8] and is also an angiogenic peptide.[9]

During studies with the Langendorff heart, it was observed that LPS acutely stimulates $Ca^{2+}$ entry in cardiomyocytes, judged by the increase of contractility and endogenous lipolysis.[10] Later it was found,[11,12] that when LPS was bound to albumin during perfusion of Langendorff hearts, it was not able to stimulate contractility or lipolysis, so that *in vivo*, direct effects of LPS cannot be important for the toxic syndrome, but only after its induction of TNF and platelet activating factor (PAF) production.[13] That TNF plays a central role in the pathogenesis of septic shock has been shown by a number of groups.[14–17]

### Involvement of Endothelium

From the brief introduction, it is clear that endothelial cells play an early and decisive role in the pathophysiology of shock. These cells are very vulnerable as they are directly exposed to the circulating blood, and moreover readily suffer from (imminent) ischaemia as will be discussed below.

The introduction mentioned the findings of others on adhesion of cells from the blood to endothelial cells as leucocytes and thrombocytes. This is accompanied by a rise of intra-endothelial $Ca^{2+}$, which stimulates phospholipase $A_2$. It results in arachidonic acid (and lysophospholipid) release from membrane phospholipids, which allows the synthesis of various autacoids. One of the endothelial prostaglandins is prostacyclin.[18] This $PGI_2$ only affects local phenomena, such as inhibition of platelet aggregation. When endothelial cells are not activated they do not cause platelet aggregation as they have a non-thrombogenic surface (a negatively charged glycocalyx containing

© 1993 Rapid Communications of Oxford Ltd glycoproteins and proteoglycans, enriched with heparan- and chondroitin sulphates). Upon surface damage platelets adhere and aggregate. The anti-aggregatory effects of $PGI_2$ is due to increase of cAMP in platelets which lowers their intracellular $Ca^{2+}$ level, which makes the platelet insensitive to activation for aggregation. After exposure to TNF (and/or PAF) activation of cyclooxygenase rapidly takes place.[19] Whereas $PGI_2$ is the most potent anti-aggregative agent, platelets make an aggregation promoting thromboxan, $TXA_2$, from arachidonic acid. The ratio $TXA_2/PGI_2$ therefore is an important determinant for aggregation. Endothelium derived relaxing factor (EDRF, i.e. the free radical NO), increases cGMP in platelets, which like cAMP decreases its $Ca^{2+}$ level and inhibits aggregation. Other endothelial compounds of great interest are endothelins-1, -2 and -3. They are vasoconstrictors, consisting of 21 amino acids and derived from 'big-endothelin'.[20] As there is no stock of the endothelins in the cells, it is the converting enzyme that determines their activity. ET-1 is the strongest vasoconstrictor and ET-3 the weakest, but the strongest aggregator of thrombocytes. Increased levels of endothelins have been found in septic shock, myocardial infarction and diabetes. Activation of endothelial cells (e.g. by thrombin) induces the synthesis of platelet activating factor (PAF or 1-alkyl-2-acetyl-glycero-3-phosphorylcholine).[21] *In vitro*, it could activate platelet aggregation. Whether this also holds for *in vivo* situations is not certain as it remains within the cells. PAF, like lysolecithin,[22] affects the cell surface.

### Vulnerability of Vascular Endothelium and Loss of Carnitine

Earlier, it was observed that the vascular surface continuously produces lysophospholipids during cell free Langendorff perfusion of control rat hearts.[23] This process is stimulated by stress hormones like glucagon and noradrenaline Therefore, continuous repair of the phospholipid bilayer by (activated) fatty acids is required. Hence the well-known stabilizing effect of palmitoylcarnitine might partly be due to this process and partly to the ability of this amphiphilic compound to affect membrane fluidity. This stabilization of the cell surface is even more urgent when tissues are subject to hypoperfusion when the equilibrium between synthesis and breakdown is disturbed. Endothelial cells, in particular, are sensitive to hypoperfusion as they suffer from their high content of xanthine dehydrogenase, which upon conversion to xanthine oxidase may generate (damaging) oxygen free radicals.[24,25]

### Effect of Carnitine Supply on Endothelial Functions

Endothelial cells catalyse carnitine dependent fatty acid oxidation,[26] so that they are able to generate long-chain acyl CoA and acyl carnitine. That endothelium is most vulnerable to hypoperfusion, as mentioned above, may result in preferential loss of low molecular components, like carnitine, from these cells. Indeed, during reperfusion after ischaemia of Langendorff rat hearts, a loss of carnitine from a non-myocyte compartment was observed, probably vascular endothelial cells.[27] It may explain the restoration of flow regulation by carnitine addition in imminent ischaemia, as recently reviewed.[27,28] Oxygen free radicals might be involved in the loss of carnitine from endothelium, as observed after reperfusion following ischaemia.[27] Therefore, carnitine supply may prevent the loss of flow regulation by endothelium in imminent ischaemia. Such a situation may be the early stage of endotoxin shock when capillary flow in many organs becomes endangered as can be demonstrated *ex vivo*, after injection of rats with LPS.[11] After 3 h the animals showed signs of dysthermia, lethargy and diarrhoea. Yet their hearts, tested *in vitro* during Langendorff perfusion, did not show contractile failure or lower lipoprotein lipase activity, indicating intactness of the cardiomyocytes. However, the microcirculation was affected, judged by an increase of cardiac interstitial fluid production from $1.08 \pm 0.19\%$ (control, $n = 6$) to $12.6 \pm 2.7\%$ (LPS rats, $n = 6$) of the total effluent, as described previously.[11] The *in vitro* perfusion of hearts from control animals for 3 h with TNF affected neither lipoprotein lipase activity[11] nor interstitial fluid production.[11] The latter is in agreement with the conclusions reached by Langeler *et al.*[29] based upon studies with an *in vitro* model of endothelial monolayers. Therefore, it is not likely that TNF directly affects increased permeability, but another product of LPS toxicity, such as PAF. Preliminary experiments indicated that 10 nM PAF increased interstitial fluid secretion during 3 h Langendorff perfusion of hearts from control rats to $17.3 \pm 2.9\%$ ($n = 3$) of the total effluent. Effects of carnitine and derivatives upon this phenomenon may be expected since Van Hinsbergh *et al.*[30] observed that propionyl carnitine decreases intracellular $Ca^{2+}$ levels in thrombin challenged endothelial cells in culture. PAF has also been shown to increase intracellular $Ca^{2+}$ after specific binding to isolated endothelial cells,[31,32] so that carnitine and carnitine derivatives may be expected to antagonize PAF effects.

### References

1. Beutler B, Greenwald D, Hulmes JD, *et al.* Identity of tumor necrosis factor and the macrophage-secreted factor cachectin. *Nature* (Lond) 1985; **316**: 552 554.

2. Pennica D, Nedwin GE, Hayflick JS, *et al.* Human tumour necrosis factor: precursor structure, expression and homology to lymphotoxin. *Nature* (Lond) 1985; **312**: 724–729.
3. Beutler B, Cerami A. Cachectin and tumour necrosis factor as two sides of the same biological coin. *Nature* (Lond) 1986; **320**: 584–588.
4. Beutler B, Mahoney J, LeTrang N, Pekala P, Cerami A. Purification of cachectina lipoprotein lipase suppressing hormone secreted by endotoxin induced RAW 246.7 cells. *J Exp Med* 1985; **161**: 984–995.
5. Bevilacqua MP, Pober JS, Majeau GR, Fiers W, Cotran RS, Gimbrone Jr, MA. Recombinant tumor necrosis factor induces procoagulant activity in cultured human vascular endothelium: characterization and comparison with the actions of interleukin 1. *Proc Natl Acad Sci* (*USA*) 1986; **83**: 4533–4537.
6. Clarke MA, Chen MJ, Crooke ST, Bomalaski JS. Tumour necrosis factor (cachectin) induces phospholipase $A_2$ activity and synthesis of a phospholipase $A_2$ activating protein in endothelial cells. *Biochem J* 1988; **250**: 125–132.
7. Nawroth PP, Stern DM. Modulation of endothelial cell hemostatic properties by tumour necrosis factor. *J Exp Med* 1986; **163**: 740–745.
8. Ward PA. Mechanism for endothelial injury. *J Lab Clin Med* 1991; **118**: 421–426.
9. Risau W, Hallmann R, Gautschi P, Bohlen P. Tumour necrosis factor type α, a potent inhibitor of endothelial cell growth *in vitro*, is angiogenic *in vivo*. *Proc Natl Acad Sci* (*USA*) 1987; **84**: 5277–5281.
10. Hülsmann WC, Lamers JMJ, Stam H, Breeman WAP. Calcium overload in endotoxemia. *Life Sci* 1981; **10**: 1009–1014.
11. Hülsmann WC, Dubelaar ML, De Witt LEA, Persoon NLM. Cardiac lipoprotein lipase: effects of lipopolysaccharide and tumour necrosis factor. *Mol Cell Biochem* 1988; **79**: 137–145.
12. Hülsmann WC, Dubelaar ML. Effect of tumour necrosis factor (TNF) on lipolytic activities of rat heart. *Mol Cell Biochem* 1988; **79**: 147–151.
13. Romano M, Molino M, Cerletti C. Endotoxic lipid-A induces intracellular $Ca^{2+}$ increase in human platelets. *Biochem J* 1991; **278**: 75.
14. Glauser MP, Zanetti G, Baumgartner JD, Cohen J. Septic shock pathogenesis. *Lancet* 1991; **338**: 732–736.
15. Waage A, Halstensen A, Espevik T. Association between tumour necrosis factor in serum and fatal outcome in patients with meningococcal disease. *Lancet* 1987; **i**: 355–357.
16. Exley AR, Cohen J, Buurman WA, *et al.* Monoclonal antibody to TNF in severe septic shock. *Lancet* 1990; **335**: 1275–1277.
17. Vanderpoll T, Romijn JA, Endert E, Borm JJJ, Bueller HR, Sauerwein HP. Tumour necrosis factor mimics the metabolic response to acute infection in healthy humans. *Am J Physiol* 1991; **261**: E457–E465.
18. Moncada S, Gryglewski R, Bunting S, Vane JR. An enzyme isolated from arteries transforming prostaglandin endoperoxides to an unstable substance that inhibits platelet aggregation. *Nature* 1976; **263**: 663–665.
19. Brevario F, Proserpio P, Bertocchi F, Lampugnani MG, Montovani A, Dejana E. Interleukin-1 stimulates prostacyclin production by cultured human endothelial cells by increasing arachidonic acid mobilization and conversion. *Arteriosclerosis* 1990; **10**: 129–134.
20. Anggard EE. The endotheliium—the body's largest endocrine gland. *J Endocrin* 1990; **127**: 371–375.
21. Prescott SM, Zimmerman GA, McIntyre TM. Human endothelial cells in culture produce platelet-activating factor (1-alkyl-2-acetyl-sn-glycero-3-phosphocholine) when stimulated with thrombin. *Proc Natl Acad Sci* (*USA*) 1984; **81**: 3534–3538.
22. Vanderbend RL, Dewidt J, Vancorven EJ, Moolenaar WH, Vanblitterswijk WJ. The biologically active phospholipid, lysophosphatidic acid, induces phosphatidylcholine breakdown in fibroblasts via activation of phospholipase-D—comparison with the response to endothelin. *Biochem J* 1992; **285**: 235–240.
23. Stam H, Hülsmann WC. Release of lipolytic products from rat heart. Hormonal stimulation, intracardiac origin and pharmacological modification. *Bioch Int* 1981; **2**: 477–484.
24. Hülsmann WC, Dubelaar ML. Early damage of vascular endothelium during cardiac ischaemia. *Cardiovasc Res* 1987; **21**: 674–677.
25. Geeraerts MD, Ronveaux-Dupal MF, Lemasters JJ, Herman B. Cystolic free $Ca^{2+}$ and proteolysis in lethal oxidative injury in endothelial cells. *Am J Physiol* 1991; **261**: C889–C896.
26. Hülsmann WC, Dubelaar ML. Aspects of fatty acid metabolism in vascular endothelial cells. *Biochemie* 1988; **70**: 681–686.
27. Hülsmann WC, Dubelaar ML. Carnitine requirement of vascular endothelial and smooth muscle cells in imminent ischaemia. *Mol Cell Biochem* 1992; **116**: 125–129.
28. Hülsmann WC, Dubelaar ML. Carnitine in metabolism of paced cardiac and skeletal muscle; prevention of acidosis and improvement of vascular flow. In: Ferrari R, Di Mauro S, Sherwood G, eds. *L-Carnitine and its role in medicine, from function to therapy*. Academic Press (London) 1992; 345–358.
29. Langeler EG, Fiers W, Van Hinsberg VWM. Effects of tumor necrosis factor on prostacyclin production and the barrier function of human endothelial cell monolayers. *Arteriosclerosis Thrombosis* 1991; **11**: 872–881.
30. Van Hinsberg VWM, Scheffer MA. Effect propionyl-L-carnitine in human endothelial cells. *Cardiovasc Drugs Ther* 1991; **5**: 97–106.
31. Hirafuji M, Shinoda H. Antagonism of PAF-induced increase in cytosolic free calcium concentration in human endothelial cells. *Japan J Pharmacol* 1992; **58**: 231–241.
32. Shukla SD. Platelet-activing factor receptor and signal transduction mechanisms. *FASEB J* 1992; **6**: 2296–2301.
